# Deficiency of Intellectual Disability-Related Gene *Brpf1* Attenuated Hippocampal Excitatory Synaptic Transmission and Impaired Spatial Learning and Memory Ability

**DOI:** 10.3389/fcell.2021.711792

**Published:** 2021-08-17

**Authors:** Weiwei Xian, Jingli Cao, Xiangshan Yuan, Guoxiang Wang, Qiuyan Jin, Hang Zhang, Guomin Zhou, Linya You

**Affiliations:** ^1^Department of Human Anatomy and Histoembryology, School of Basic Medical Sciences, Fudan University, Shanghai, China; ^2^Institutes of Brain Sciences, Fudan University, Shanghai, China; ^3^Key Laboratory of Medical Imaging Computing and Computer Assisted Intervention of Shanghai, Shanghai, China

**Keywords:** intellectual disability, BRPF1, primary cultured hippocampal neurons, dendritic morphology, electrophysiology, stereotactic hippocampal injection, Morris water maze, mRNA-seq

## Abstract

Patients with monoallelic bromodomain and PHD finger-containing protein 1 (*BRPF1*) mutations showed intellectual disability. The hippocampus has essential roles in learning and memory. Our previous work indicated that *Brpf1* was specifically and strongly expressed in the hippocampus from the perinatal period to adulthood. We hypothesized that mouse *Brpf1* plays critical roles in the morphology and function of hippocampal neurons, and its deficiency leads to learning and memory deficits. To test this, we performed immunofluorescence, whole-cell patch clamp, and mRNA-Seq on shBrpf1-infected primary cultured hippocampal neurons to study the effect of *Brpf1* knockdown on neuronal morphology, electrophysiological characteristics, and gene regulation. In addition, we performed stereotactic injection into adult mouse hippocampus to knock down *Brpf1 in vivo* and examined the learning and memory ability by Morris water maze. We found that mild knockdown of *Brpf1* reduced mEPSC frequency of cultured hippocampal neurons, before any significant changes of dendritic morphology showed. We also found that *Brpf1* mild knockdown in the hippocampus showed a decreasing trend on the spatial learning and memory ability of mice. Finally, mRNA-Seq analyses showed that genes related to learning, memory, and synaptic transmission (such as *C1ql1*, *Gpr17*, *Htr1d*, *Glra1*, *Cxcl10*, and *Grin2a*) were dysregulated upon *Brpf1* knockdown. Our results showed that *Brpf1* mild knockdown attenuated hippocampal excitatory synaptic transmission and reduced spatial learning and memory ability, which helps explain the symptoms of patients with *BRPF1* mutations.

## Introduction

Intellectual disability is a prevalent neurodevelopmental disorder, affecting 1–2% of children or young adults. Over 1,396 genes have been implicated in intellectual disability (Ilyas et al., 2020). Bromodomain and PHD finger-containing protein 1 (*BRPF1*) is an important candidate gene with its monoallelic mutations resulting in intellectual disability (Mattioli et al., 2017; Yan et al., 2017; Demeulenaere et al., 2019; Pode-Shakked et al., 2019), but the exact underlying mechanism remains unclear.

BRPF1 is a chromatin regulator with multiple modules, including double PHD fingers (recognizing the N-terminal tail of histone H3) (Poplawski et al., 2014; Klein et al., 2016), a bromodomain (with acetyl-lysine-binding ability) (Lubula et al., 2014b), and a PWWP domain (forming a specific pocket for trimethylated histone H3) (Vezzoli et al., 2010). BRPF1 acts as a scaffold to promote the binding of transcription factors to the acetyltransferase MOZ/MORF and activate acetylation of histones H2A, H2B, H3, and H4 (Lubula et al., 2014a). The cumulative number of patients with *BRPF1* mutations has reached 40, and patients were often accompanied with symptoms such as epilepsy, hypotonia, developmental delay, language dysfunction, ptosis, and cerebellar and facial deformities (Mattioli et al., 2017; Yan et al., 2017, 2020; Demeulenaere et al., 2019; Pode-Shakked et al., 2019). The expression level of *BRPF1* was positively correlated with the volume of hippocampal CA2, CA3, and dentate gyrus (Zhao et al., 2019). Our previous studies indicated that mouse *Brpf1* is strongly and specifically expressed in the hippocampus during fetal, neonatal, and adult stages (You et al., 2014, 2015a). Similarly, in human, *BRPF1* is also stably expressed in the hippocampus from development to adulthood by BrainSpan Atlas, a transcriptional resource of the developing human brain (Zhao et al., 2019).

Our previous studies showed that homozygous deletion of *Brpf1* led to embryonic lethality, and one of the defects was in neural tube closure (You et al., 2014, 2015a). Forebrain-specific deletion of mouse *Brpf1* resulted in early postnatal lethality with neocortical abnormalities and partial agenesis of the hippocampus and corpus callosum (You et al., 2015b,c). Another group showed that forebrain-specific heterozygous deletion of *Brpf1* exhibited decreased the ability of learning and memory and reduced the dendritic complexity and density of spinous processes (Su et al., 2019).

The hippocampus has essential roles in learning and memory. CA1 pyramidal neurons play an important role in long-term memory and spatial-related tasks (Ferguson et al., 2004). Our previous study showed that *Brpf1* was specifically and strongly expressed in the hippocampus, especially CA1 and dentate gyrus (You et al., 2015b). We hypothesized that *Brpf1* regulates the morphology and function of hippocampal neurons, and dysfunction of *Brpf1* eventually leads to impaired learning and memory. In this study, we examined the effect of *Brpf1* knockdown on neuronal morphology, electrophysiological characteristics, and gene regulation by immunofluorescence, whole-cell patch clamp, and mRNA-Seq, respectively, using shRNA-infected primary cultured hippocampal neurons. In addition, we evaluated the impact of *Brpf1* deficiency on learning and memory ability by Morris water maze using shRNA stereotactic injection into adult mouse hippocampus. Our findings indicated that *Brpf1* mild knockdown attenuated hippocampal excitatory synaptic transmission and reduced spatial learning and memory ability, which helps explain the symptoms of patients with *BRPF1* mutations.

## Materials and Methods

### Animals

All mice used were of C57BL/6 background purchased from Jiesijie lab (Shanghai, China). Animals were maintained on a 12-h light/dark cycle. The day when vaginal plug is detected was considered to be embryonic day 0.5 (E0.5). All animal experiments were approved by the local committees of the guidelines of the laboratory animals at Fudan University (Shanghai, China).

### Hippocampal Primary Neuronal Cultures and Adeno-Associated Virus (AAV) Infection

Primary hippocampal neurons were generated from E17.5 to E18.5 mouse embryos as described previously (Beaudoin et al., 2012). Briefly, hippocampi from E17.5 to E18.5 embryos were dissected in HBSS and dissociated in 0.25% of trypsin. The dissociated cells were plated onto poly-L-lysine-coated 24-well plates with neurobasal medium supplemented with B27 supplement (17504044, Gibco, New York, NY, United States). Media were changed twice a week. To knock down *Brpf1*, cultured neurons at days *in vitro* (DIV) 3 were infected with either AAV2-scramble-green fluorescent protein (GFP) or AAV2-shBrpf1-GFP purchased from BrainVTA (Wuhan, China). The sense sequence of shBrpf1 is GGCTTACCGCTACTTGAACTT. Media were replaced after 12 h. Experiments using primary cultured neurons were performed at DIV 14–15. Neurons were grown at 37∘C and with 5% CO_2_.

### Stereotactic Viral Vector Delivery

4-week-old mice were fixed on a stereotactic device and anesthetized with isoflurane inhalation during the surgery. AAV2-scramble-GFP and AAV2-shBrpf1-GFP were injected into the left and right hippocampal CA1 through a glass micropipette at a rate of 0.02 μl/min, respectively. The total volume of AAVs injected into each hemisphere was 0.2 μl. The glass microelectrode was kept in the CA1 for 5 min to allow the AAVs to spread. The stereotactic coordinates of CA1 are anterior–posterior to bregma: –1.8 mm, middle-lateral to middle line: ± 1.2 mm, dorsal-ventral to cerebral dura mater: ± 1.2 mm. The mice then underwent behavioral or molecular experiments 4 weeks after operation.

### Morris Water Maze

Mice after stereotactic operation were introduced into a circular water-filled tank 120 cm in diameter with visual cues that were present on the tank walls as spatial references. The temperature of water was maintained at 22.0°C, and white non-toxic paint was used to make the water turbid and opaque. The tank was divided into northwest (NW), northeast (NE), southwest (SW), and southeast (SE) quadrants by lines. A round platform with 10 cm of diameter was submerged 1 cm deep in the tank. On day 1, mice were put in a room for at least 30 min to be familiar with the environment before training. Each mouse was put gently into the water with the head faced to the wall of the tank during training, given 60 s to find the platform, and allowed to stay on the platform for 15 s when the mouse reached the platform. The experimenter guided it to the platform and let it stay on the platform for 15 s if the mouse could not find the platform within 60 s. Each mouse received four trials daily from four different quadrants. On day 8, the mice were subject to a single 60-s probe trial without a platform to test memory retention. EthoVision (Noldus, Wageningen, Netherlands) was used to track the mice and analyze the data.

### Immunofluorescence Staining and Analysis of Dendritic Morphology

Neurons on coverslips were fixed with 4% of paraformaldehyde for 20 min at room temperature, blocked with 5% of goat serum and 0.1% of Triton X-100 in PBS for 1 h, incubated overnight at 4°C with mouse anti-MAP2 (67015-1-ig, 1:2,000, Proteintech, Wuhan, China) and rabbit anti-GFP (Proteintech, 50430-2-AP, 1:500), and then incubated with Alexa fluorophore-labeled secondary antibodies (goat anti-rabbit 488 and goat anti-mouse 594) for 1 h at room temperature. Finally, the neurons were counterstained with DAPI (2 μg/ml) for 5 min and mounted with fluorescence mounting medium. Images were acquired using a confocal microscope (Leica SP8) and analyzed by ImageJ Fiji. Sholl analysis was applied to calculate the branches and the total length of dendrites (O’Neill et al., 2015).

### Reverse Transcription and Quantitative Real-Time PCR

Total RNA was extracted from cultured hippocampal neurons or hippocampal CA1 tissue by the TRIzol method. 500 ng of RNA of each sample was used for reverse transcription with Evo M-MLV RT Kit with gDNA Clean (Accurate Biotechnology, Hunan, China, AG11705). The resulting cDNA was used as a template for RT-qPCR using TB Green Premix Ex Taq (Takara, Shiga, Japan, rr420a). *GAPDH* was used as the internal control, and the relative expression of the gene was calculated by the 2^–ΔΔCt^ method (Livak and Schmittgen, 2001). The primer list is summarized in Supplementary Table 1.

### RNA Sequencing and Analysis

Total RNA was extracted from three pairs of primary cultured hippocampal neurons and three pairs of stereotactic injected CA1 tissues with RIN values of more than 9. The samples were subject to high-throughput mRNA sequencing through paired-end mode with the Illumina HiSeq sequencing platform. For data analysis, the preprocessing sequence was compared with the mouse genome (release-98) by STAR software after removing the linker and low-quality fragments. StringTie software was used to count the original sequence counts of known genes, and the expression of known genes was calculated using fragments per kilobase of transcript per million fragments mapped (FPKM). The DESeq2 software was applied to screen the differentially expressed genes (DEGs) between different groups [log_2_ (fold change) ≥ 1 or ≤ –1 and *p*-value < 0.05 was used]. The function of DEGs was analyzed by the David platform (Huang Da et al., 2009a,b), including gene ontology (GO) and Kyoto Encyclopedia of Genes and Genomes (KEGG). The raw and processed data were deposited in the Gene Expression Omnibus database with GEO# GSE174600.

### Electrophysiology Recording and Analysis

For whole-cell patch-clamp recording, neurons were infected with a reduced titer of AAV2-scramble-GFP or AAV2-shBrpf1-GFP at DIV3 to achieve sparse labeling, and the recordings were performed at DIV15. Whole-cell recordings were conducted with a conventional patch-clamp technique with a MultiClamp 700B amplifier (Axon Instruments, Burlingame, CA, United States). Patch pipettes were pulled by a Sutter P-97 pipette puller (Sutter Instrument, Novato, CA, United States). The bath solution (pH = 7.3) contained 128 mM NaCl, 5 mM KCl, 25 mM HEPES, 1 mM MgCl_2_, 30 mM glucose, and 2 mM CaCl_2_. Recording pipettes were filled with an internal solution (pH = 7.2–7.3) containing 125 mM potassium gluconate, 10 mM KCl, 10 mM HEPES, 5 mM EGTA, 10 mM Tris–phosphocreatine, 4 mM MgATP, and 0.5 mM Na_2_GTP. All the recordings were performed at room temperature. Data acquisition and analysis were performed with pClamp 10.2 software (Axon Instruments, Burlingame, CA, United States).

### Statistics

For two-group comparisons, two-tailed and unpaired Student’s *t*-tests were performed. All statistical analyses were performed using Prism 7 software (GraphPad). The value of *p* < 0.05 was considered statistically significant. ^∗^*p* < 0.05, ^∗∗^*p* < 0.01, and ^∗∗∗^*p* < 0.001.

## Results

### Fifty Percent Knockdown of *Brpf1* Did Not Significantly Change the Dendritic Length and Number of Intersections *in vitro*

**FIGURE 1:**
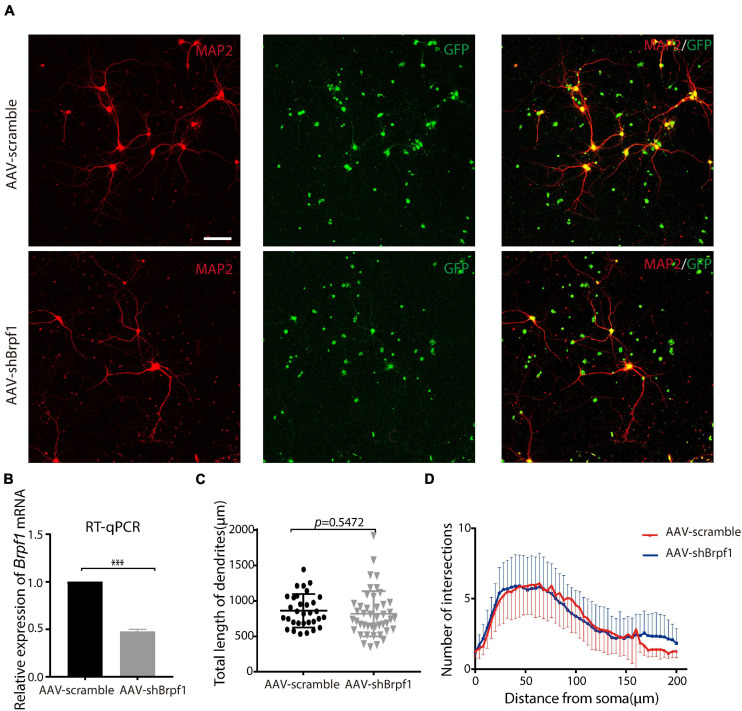
Mild knockdown of *Brpf1* did not significantly change the total dendritic length and number of intersections. **(A)** Representative fluorescent images of cultured primary hippocampal neurons at DIV15 immunostained with anti-Map2 antibody, scale bar = 75 μm. **(B)** Quantitative mRNA analysis of *Brpf1* knockdown efficiency (*n* = 4 batches for AAV-scramble and AAV-shBrpf1 groups, respectively, *p* < 0.001). **(C)** Quantification of total dendritic length of AAV-scramble or AAV-shBrpf1 group using “Simple Neurite Tracer” of ImageJ (*n* = 32 or 44 neurons from three independent batches for AAV-scramble or AAV-shBrpf1 group, respectively, *p* = 0.5472). **(D)** Sholl analysis of the number of intersections of the dendrites (*n* = 32 or 44 neurons from three independent batches for AAV-scramble or AAV-shBrpf1 group, respectively). ***p < 0.001.

Neurons have unique dendritic-like structures, which play an important role in signal transmission. To study the effect of *Brpf1* knockdown on the dendritic morphology of hippocampal neurons, we dissociated E17.5–E18.5 hippocampi and infected primary cultured neurons with AAV2-scramble-GFP or AAV2-shBrpf1-GFP at DIV3. Immunostaining was performed at DIV 14–15 with anti-MAP2 antibody (a specific marker for dendrites), and the quantification of dendritic morphology was calculated from three independent experiments using Sholl analysis (O’Neill et al., 2015); totally 32 and 44 neurons in the scramble and shBrpf1 groups were analyzed, respectively (Figure 1A). We confirmed a knockdown efficiency of approximately 50% by RT-qPCR (Figure 1B). The results showed that the average total dendritic length and number of intersections of primary cultured hippocampal neurons did not change significantly upon half reduction of *Brpf1* expression (Figures 1C,D).

### Fifty Percent Knockdown of *Brpf1* Led to Decreased mEPSC Frequency *in vitro*

**FIGURE 2:**
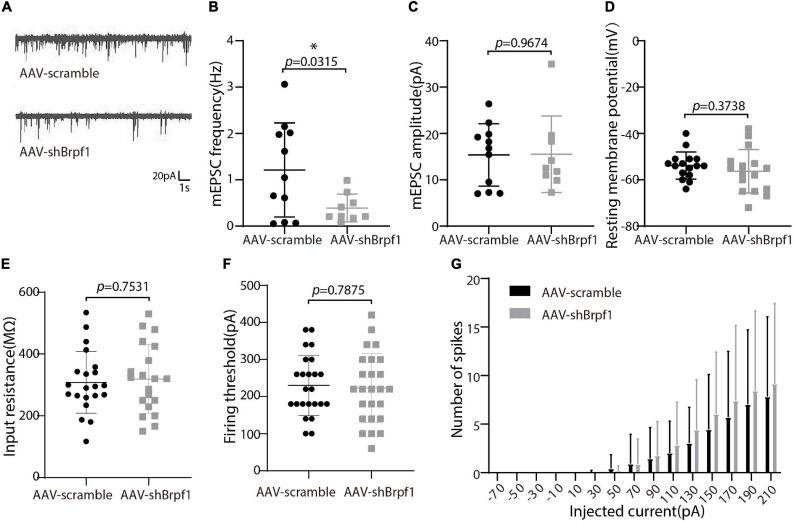
Mild knockdown of *Brpf1* led to decreased mEPSC frequency. **(A)** Representative traces of mEPSCs recorded in cultured primary hippocampal neurons of AAV-scramble or AAV-shBrpf1 group, scale bar, 20 pA and 1 s. **(B)** Statistical comparison of mEPSC frequency between AAV-scramble and AAV-shBrpf1 groups (AAV-scramble, *n* = 11 neurons; AAV-shBrpf1, *n* = 9 neurons; *p* = 0.0315). **(C)** Statistical comparison of mEPSC amplitude using the same dataset as **(B)** (*p* = 0.9674). **(D)** Comparison of RMP between the two groups (AAV-scramble, *n* = 16 neurons; AAV-shBrpf1, *n* = 16 neurons; *p* = 0.3738). **(E)** Comparison of input resistance (AAV-scramble, *n* = 21 neurons; AAV-shBrpf1, *n* = 20 neurons; *p* = 0.7531). **(F)** Comparison of firing threshold (AAV-scramble, *n* = 25 neurons; AAV-shBrpf1, *n* = 24 neurons; *p* = 0.7875). **(G)** The number of spikes displayed against depolarizing current steps of increasing amplitude with injected currents ranging from –70 to 210 pA at 20-pA intervals (AAV-scramble, *n* = 25 neurons; AAV-shBrpf1, *n* = 24 neurons). *p < 0.05.

Alterations in learning and memory are closely associated with synaptic dysfunction (Volk et al., 2015). To study the synaptic transmission of hippocampal neurons upon *Brpf1* knockdown, we performed whole-cell patch clamp recordings on cultured hippocampal neurons at DIV 15 after scramble or shBrpf1 infection at DIV3 (Figure 2). Because most hippocampal neurons are excitatory neurons, we recorded miniature excitatory postsynaptic currents (mEPSCs). We recorded 6 min and selected the last 3 min for analysis because signals from the first 3 min were less stable. The results showed that the mEPSC frequency but not amplitude significantly decreased upon *Brpf1* knockdown (Figures 2A–C), suggesting reduced excitatory synaptic transmission. For cell membrane characteristics, we measured resting membrane potential (RMP) and evoked action potentials (APs). The RMP, input resistance, firing threshold, and evoked APs did not show significant changes (Figures 2D–G), indicating few changes of cell excitability. Collectively, these results suggested that 50% knockdown of *Brpf1* already led to reduced synaptic transmission before any obvious morphological changes.

### *Brpf1* Mild Knockdown Led to Downregulation of *C1ql1* and *Gpr17 in vitro*

**FIGURE 3:**
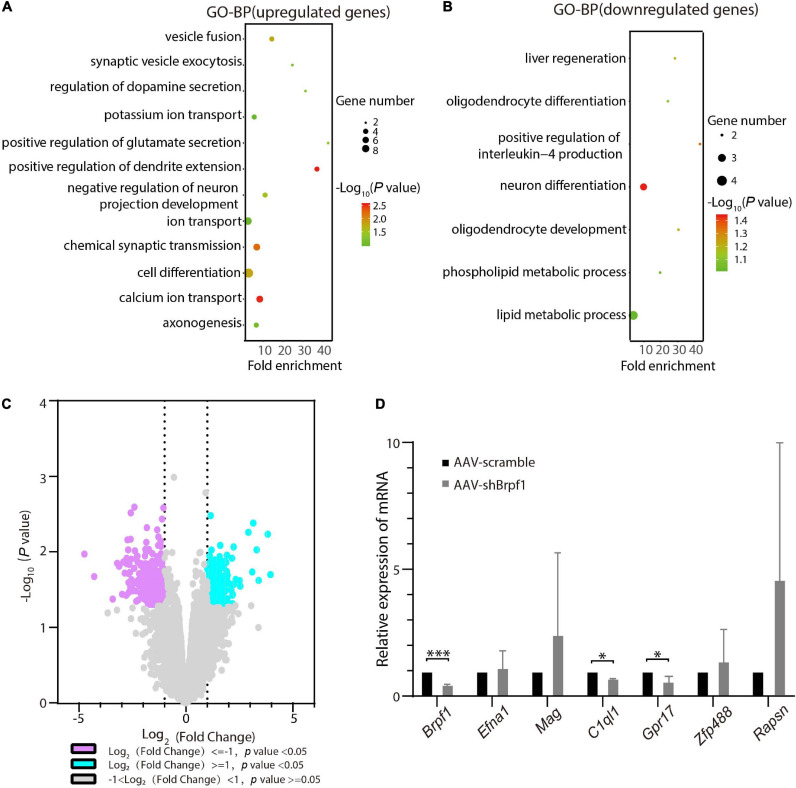
*Brpf1* knockdown led to downregulation of *C1ql1* and *Gpr17.* **(A,B)** Bubble plots represented GO-BP of upregulated genes [log_2_(fold change) ≥ 1 and *p* < 0.05] and downregulated genes [log_2_(fold change) ≤ –1 and *p* < 0.05] derived from mRNA-Seq analysis of three pairs of cultured primary hippocampal neurons, respectively. Dot size reflected gene number; color indicated –log_1__0_P. **(C)** Volcano plot of DEGs from mRNA-Seq similar to **(A,B)**. **(D)** Selected DEGs were validated by RT-qPCR (AAV-scramble group, *n* = 3 batches; AAV-shBrpf1 group, *n* = 3 batches). ^∗^*p* < 0.05, ^∗∗∗^*p* < 0.001.

To figure out the molecular mechanism of how *Brpf1* regulates the electrophysiological activity of hippocampal neurons, three batches of RNA from scramble and shBrpf1 groups were extracted for mRNA-Seq analysis. DESeq2 software was used to screen differentially expressed genes (DEGs). 218 and 402 genes were upregulated and downregulated, respectively, upon *Brpf1* knockdown (Supplementary Table 2). GO-biological process (BP) analysis revealed that upregulated genes were mainly involved in ion transport, chemical synaptic transmission, and other biological processes, while downregulated genes were mainly involved in neuronal differentiation, oligodendrocyte differentiation, and development (Figures 3A,B and Supplementary Table 3). We then selected mainly neuron-related genes for verification by RT-qPCR (Figures 3C,D). Complement C1q-like 1 (*C1ql1*) and G protein-coupled receptor 17 (*Gpr17*) were downregulated in both mRNA-Seq analysis and RT-qPCR verification upon *Brpf1* knockdown. *C1ql1* is highly expressed in the brain (Berube et al., 1999). Interestingly, *C1q* tagging of synapses plays a critical role in synaptic elimination by microglia and is seen throughout the developing brain (Stevens et al., 2007; Li and Barres, 2018). *Gpr17* plays a role in improving learning and memory in rats (Marschallinger et al., 2015). This suggested that the decreased frequency of mEPSCs caused by *Brpf1* mild knockdown may be associated with dysregulated synaptic elimination.

### *Brpf1* Mild Knockdown Showed a Tendency of Reduction on Spatial Learning and Memory Ability *in vivo*

**FIGURE 4:**
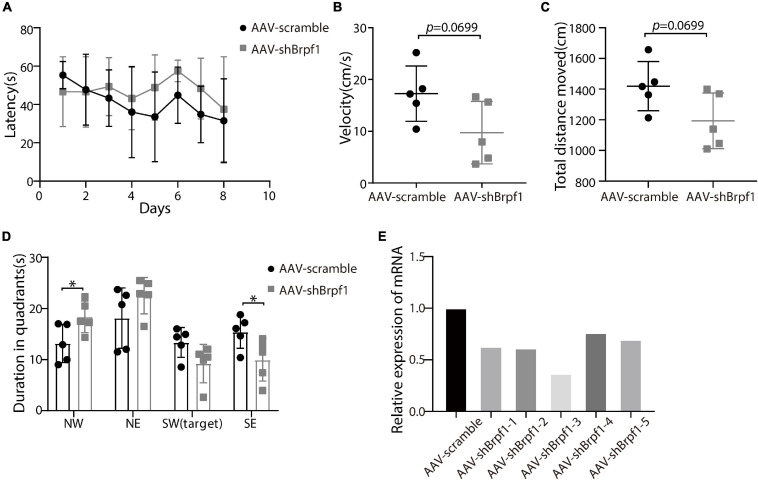
Mild knockdown of *Brpf1* showed a decreasing trend on the learning and memory ability of mice. **(A)** Statistical comparison of the latency of the mice to reach the platform during the 8-day training period (AAV-scramble, *n* = 5 mice; AAV-shBrpf1, *n* = 5 mice). **(B)** Statistical comparison of the average swimming speed of mice on day 8 in the target quadrant (AAV-scramble, *n* = 5 mice; AAV-shBrpf1, *n* = 5 mice; *p* = 0.0699). **(C)** Statistical comparison of the total moving distance of mice in the target quadrant (AAV-scramble, *n* = 5 mice; AAV-shBrpf1, *n* = 5 mice; *p* = 0.0699). **(D)** Statistical comparison of the time spent in different quadrants on day 8 (AAV-scramble, *n* = 5 mice; AAV-shBrpf1, *n* = 5 mice; ^∗^*p* < 0.05). **(E)** Relative expression of *Brpf1* mRNA in the hippocampus of each mouse compared to the scramble group.

To study whether *Brpf1* knockdown in the hippocampus affects the learning and memory ability of mice, we injected AAV2-scramble-GFP or AAV2-shBrpf1-GFP into 4-week-old mouse hippocampi by stereotactic operation. Four weeks after surgery, we performed the Morris water maze test to assess the spatial learning and memory ability of those mice. Although the latency of mice to reach the platform was almost the same (Figure 4A), a mild decreasing trend of the swimming speed and the total distance moved in the probe trail were found upon *Brpf1* knockdown (Figures 4B,C). Moreover, there was a significant difference of their stay time in the northwest (NW) and southeast (SE) quadrants, indicating a decreasing tendency of the space exploration ability, although no significant difference in the target quadrant (SW) was found (Figure 4D). This behavioral change was caused by a mild knockdown of *Brpf1*, with the knockdown efficiency varying from 40 to 70% in five mice tested (Figure 4E). These results indicated that *Brpf1* mild knockdown in the hippocampus led to a tendency of reduction on the spatial learning and memory ability.

### *Brpf1* Mild Knockdown in the Hippocampus Led to Dysregulated Gene Expression Related to Synaptic Function *in vivo*

**FIGURE 5:**
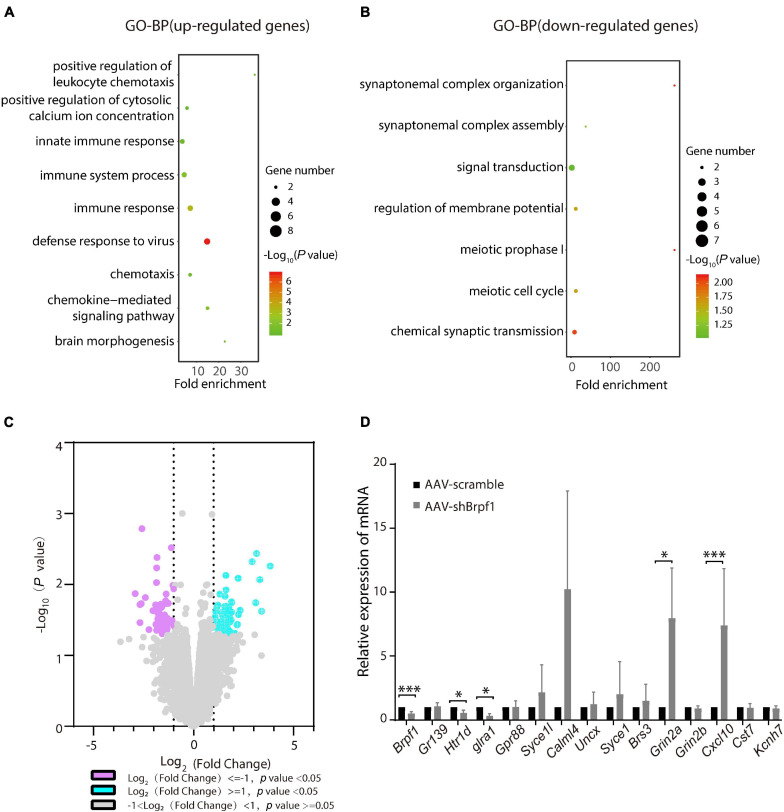
Hippocampus-specific knockdown of *Brpf1* regulated the expression of genes related to synaptic function. **(A,B)** Bubble plots represented GO-BP of upregulated genes [log_2_(fold change) ≥ 1 and *p* < 0.05] and downregulated genes [log_2_(fold change) ≤ –1 and *p* < 0.05] derived from mRNA-Seq analysis of three pairs of stereotactic injected CA1 tissue, respectively. Dot size reflected gene number; color indicated –log_1__0_P. **(C)** Volcano plot of DEGs from mRNA-Seq similar to **(A,B)**. **(D)** Selected DEGs were validated by RT-qPCR (AAV-scramble group, *n* = 3 mice; AAV-shBrpf1 group, *n* = 3 mice). ^∗^*p* < 0.05, ^∗∗∗^*p* < 0.001.

To study the molecular mechanism of *Brpf1* knockdown leading to a decreasing trend in learning and memory ability, three pairs of RNA from scramble and shBrpf1 stereotactically injected CA1 tissues were extracted for mRNA-Seq analysis. 99 upregulated and 74 downregulated genes were subject to GO-BP analysis (Supplementary Table 4). We found that upregulated genes were mainly involved in immune response, brain morphogenesis, and other processes, while downregulated genes were mainly involved in signal transduction, regulation of membrane potential, and chemical synaptic transmission (Figures 5A,B and Supplementary Table 5). We then selected genes related to learning and memory from DEGs and verified them by RT-qPCR (Figures 5C,D). The results showed that glutamate ionotropic receptor NMDA-type subunit 2A (*Grin2a*) and C–X–C motif chemokine ligand 10 (*Cxcl10*) were significantly upregulated, while 5-hydroxytryptamine receptor 1D (*Htr1d*) and glycine receptor alpha 1 (*Glra1*) were significantly downregulated. Gr*in2a*-encoded protein is a subunit of N-methyl-D-aspartate (NMDA) receptor, the activation of which results in a calcium influx into postsynaptic cells (Franchini et al., 2020). *Cxcl10* could modulate neuronal activity and plasticity in the hippocampus (Kodangattil et al., 2012). Upregulation of *Cxcl10* could occur in response to synaptic degeneration (Fuchtbauer et al., 2011). *Htr1d* regulates the release of 5-hydroxytryptamine (5-HT), one of the main neurotransmitters in the brain, and thereby affects neural activity (Weinshank et al., 1992). *Glra1* mediates postsynaptic inhibition in the spinal cord and other areas of the central nervous system (Nakahata et al., 2017). Collectively, *Brpf1* mild knockdown in the hippocampus led to dysregulated gene expression related to synaptic function.

## Discussion

Accumulating clinical studies have reported totally 40 cases of patients with *BRPF1* monoallelic mutations, with symptoms such as intellectual disability, developmental delay, and epilepsy (Mattioli et al., 2017; Yan et al., 2017, 2020; Baker et al., 2019; Pode-Shakked et al., 2019). In this study, we found that 50% knockdown of mouse *Brpf1* reduced mEPSC frequency of primary cultured hippocampal neurons *in vitro* (Figure 2), before any significant effect showed on the neuronal dendritic morphology (Figure 1). In addition, *Brpf1* mild knockdown showed a decreasing trend on the learning and memory ability of mice (Figure 4). The underlying molecular mechanism may involve dysregulated genes related to learning, memory, and synaptic transmission (Figures 3, 5).

The imbalance of excitatory/inhibitory neural circuits is closely related to diseases with learning and memory deficits. In this study, we found that *Brpf1* mild knockdown reduced the frequency but not the amplitude of mEPSCs in cultured hippocampal neurons. Similarly, another group showed that forebrain-specific heterozygous knockout of *Brpf1* caused a significant decrease in the frequency and less significant decrease in the amplitude of hippocampal mEPSCs using acute brain slices at P30 (Su et al., 2019). The discrepancy of the effect of *Brpf1* on the amplitude of mEPSCs may be due to different models (primary cultured hippocampal neurons vs. acute hippocampal slices) and ages (E17.5–E18.5 vs. P30). It could be possible that the effect of *Brpf1* haploinsufficiency (50% knockdown) on the amplitude of mEPSCs in hippocampal neurons gradually becomes obvious as mice grow, but the effect on the frequency of mEPSCs is already significant as early as E17.5–E18.5. In addition, *Brd1*+/− (also known as *Brpf2*) pyramidal neurons showed decreased frequency of spontaneous IPSCs and mIPSCs (Qvist et al., 2017a). Interestingly, *Brd1* has been implicated in the pathogenesis of schizophrenia and bipolar disorder (Severinsen et al., 2006; Fryland et al., 2016; Qvist et al., 2017a). Besides *BRPF1*, related histone acetyltransferases *MOZ* and *MORF* were also found mutated in patients with abnormal neurodevelopment and intellectual disability (Gannon et al., 2015; Tham et al., 2015; Kennedy et al., 2019; Zhang et al., 2020); however, no studies of their effect on the electrophysiology of neurons have been reported.

The mild knockdown of *Brpf1* led to significant changes in electrophysiological properties before any major influence showed in dendritic morphology. Thus, we performed mRNA-Seq analysis to mainly explain the change in electrophysiology. The analysis revealed that *C1ql1* and *Gpr17* were significantly downregulated. *C1ql1* plays an important role in the formation and maintenance of synapses in the central nervous system (Yuzaki, 2017). Relatedly, *C1q* tagging of synapses is pivotal in synaptic pruning and prevalent in the developing brain (Stevens et al., 2007; Li and Barres, 2018). Montelukast (targets leukotriene receptors GPR17 and CysLTR1) could improve learning and memory in old rats (Marschallinger et al., 2015). In addition, *Gpr17* is a functional receptor that involves the generation of outward K^+^ currents in the signal transduction pathway and can reduce neuronal overexcitement in the brain (Pugliese et al., 2009). The association of the decreased frequency of mEPSCs in primary hippocampal neurons caused by *Brpf1* mild knockdown and possible dysregulated synaptic elimination by *C1q* is an inspiring finding that merits further investigations.

A change in mEPSC frequency often indicates presynaptic release in probability alternations, whereas a change in mEPSC amplitude often indicates postsynaptic receptor function or/and number alternations. As revealed by the enrichment analysis of the DEGs from shBrpf1 vs. scramble infected primary hippocampal neurons (Supplementary Table 3), upregulated genes Cck and Syt4 were involved in “positive regulation of glutamate secretion”. Cck regulates gastric acid secretion and food intake (Miyasaka et al., 2005), while Syt4 serves as Ca^2+^ sensors in the process of vesicular trafficking and is thought to function as an inhibitor of neurotransmitter release (Littleton et al., 1999). Both do not act directly in glutamate secretion. Also, five upregulated genes, namely, Pnoc, Fgf14, Cacnb2, Sv2b, and Htr2c, were involved in “chemical synaptic transmission”. Pnoc is crucially in the neurobiological regulation of stress-coping behavior and fear (Koster et al., 1999); Fgf14 is involved in the regulation of Purkinje cell firing by altering the expression of Nav1.6 channels (Shakkottai et al., 2009); Cacnb2 variation is associated with functional connectivity in the hippocampus in bipolar disorder (Liu et al., 2019); and Sv2b deficiency did not affect glutamatergic or GABAergic transmission (Venkatesan et al., 2012). They also do not have significant roles in glutamate secretion. How *Brpf1* deficiency affects presynaptic alternations will need more investigations.

Interestingly, decreased frequency of mEPSCs caused by *Brpf1* mild knockdown was associated with a tendency of reduction on learning and memory ability in young adult mice. Patients with *BRPF1* monoallelic mutations would only have half expression of BRPF1 throughout development and into the postnatal life. Our finding was derived from acute knockdown conditions, and the effect was mild and limited. This is consistent with previous studies showing that both *Brpf2* heterozygotes and *Brpf1* forebrain-specific heterozygous knockout mice exhibited learning, memory, and cognitive dysfunction (Qvist et al., 2017b; Su et al., 2019). Our findings further supported that only half reduction of the *Brpf1* dose could affect excitatory synaptic transmission and further impair learning and memory ability.

At the molecular level, the expression of *Cxcl10* and *Grin2a* increased significantly upon *Brpf1* mild knockdown. *Grin2a* is a subunit of the NMDA receptor, which is permeable to Na^+^, K^+^, and Ca^2+^, thereby regulating signal transduction pathways (Sanders et al., 2018). NMDA receptors are involved in long-term potentiation and activation of those receptors results in a calcium influx into postsynaptic cells (Franchini et al., 2020). Patients with *GRIN2A* mutations began to develop neurological abnormalities 1 year after birth and were often accompanied with epilepsy (Pierson et al., 2014). *Cxcl10* could induce apoptosis and death of hippocampal neurons (Sui et al., 2006; van Weering et al., 2011). Acute exposure to *Cxcl10* altered neuronal signaling properties and reduced long-term potentiation in mouse adult hippocampal slices. It also altered spontaneous synaptic network activity, spike firing, and intracellular Ca^2+^ levels in cultured hippocampal neurons (Nelson and Gruol, 2004; Vlkolinsky et al., 2004; Cho et al., 2009). In addition, *Glra1* and *Htr1d* were significantly downregulated upon *Brpf1* mild knockdown (Figure 5D). *Glra1* homozygous mutant mice showed complex motor dysfunction (Kling et al., 1997), and there was no glycine-induced current and IPSC in all bipolar cells of *Glra1*-deficient mice (Ivanova et al., 2006). *Htr1d* regulates the release of 5-HT, one of the main neurotransmitters in the brain, and thereby affects neural activity (Weinshank et al., 1992). Its mutations often led to attention-deficit/hyperactivity disorder (Li et al., 2006). Collectively, *Brpf1* mild knockdown in the hippocampus *in vivo* led to dysregulated gene expression related to synaptic function. A more direct link between these genes and *Brpf1* merits further investigations.

In summary, our results indicated that *Brpf1* plays an important role in mouse hippocampal neurons, including attenuation of electrophysiological activity, impairment of learning and memory, and changes in gene regulation, which might partially explain the mechanism of *BRPF1* mutations causing intellectual disability in children.

## Data Availability Statement

The original contributions presented in the study are publicly available. This data can be found here: Gene expression data are available at GEO with the accession number: GSE174600 (https://www.ncbi.nlm.nih.gov/geo/query/acc.cgi?acc=GSE174600).

## Ethics Statement

The animal study was reviewed and approved by local committees of the guidelines of the laboratory animals at Fudan University (Shanghai, China).

## Author Contributions

WX initiated and performed most of the experiments. JC finished the project. XY performed the stereotactic injections and helped analyze the data. WX, JC, and XY designed the experiments and analyzed the data. GW, QJ, and HZ helped collect and analyze the data. WX, JC, and LY co-wrote the manuscript. GZ and LY conceived the idea and supervised the study. All the authors contributed to the article and approved the submitted version.

## Conflict of Interest

The authors declare that the research was conducted in the absence of any commercial or financial relationships that could be construed as a potential conflict of interest.

## Publisher’s Note

All claims expressed in this article are solely those of the authors and do not necessarily represent those of their affiliated organizations, or those of the publisher, the editors and the reviewers. Any product that may be evaluated in this article, or claim that may be made by its manufacturer, is not guaranteed or endorsed by the publisher.
