# Supplementary material for: Deficiency of Intellectual Disability-Related Gene Brpf1 Attenuated Hippocampal Excitatory Synaptic Transmission and Impaired Spatial Learning and Memory Ability
Source: Front Cell Dev Biol. 2021 Aug 17;9:711792. doi: 10.3389/fcell.2021.711792 (PMC8415984; doi:10.3389/fcell.2021.711792)
Supplement: Supplementary file 1 [file Table_1.docx]

Table S1 Primer sequence

| Primer name | | Primer sequence |
| --- | --- | --- |
| shRNA | sense | GGCTTACCGCTACTTGAACTT |
|  | antisense | AAGTTCAAGTAGCGGTAAGCC |
| GAPDH | F | AGGTCGGTGTGAACGGATTTG |
|  | R | TGTAGACCATGTAGTTGAGGTCA |
| mBrpf1 | F | ATGCAGCTGACCCCTTTCCT |
|  | R | CACTGCTGCCCTGTAGAAGA |
| *Gpr139* | F | CCATTACCTCCATCTTCGCCA |
|  | R | CCAGCCAAGGGTTCTGGAT |
| *Htr1d* | F | CATCCCTCCGCTCTTCTGG |
|  | R | AGCCTGTGATAAGCTGTGCC |
| *Glra1* | F | CCTCCCTACCCAAGGTGTC |
|  | R | ATGGGGCTCTTGTGATGTCG |
| *Gpr88* | F | TCCTCCACTTCGACCTCCAC |
|  | R | GCCCGAGTACAGGAGAGAC |
| *Calml4* | F | TCTTCTGGTGTCCATGAGGTG |
|  | R | GAAGTCCAGCTCTCCGTTCTT |
| *Uncx* | F | ACCCGCACCAACTTTACCG |
|  | R | TGAACTCGGGACTCGACCA |
| *Syce1l* | F | CCAGATTGAGGACCTGATCCAC |
|  | R | TTCCTGGAGTGCTTGAGCCTCT |
| *Syce1* | F | AGACTGTCTGGGACAACCTGCA |
|  | R | GTGCCTCACTTTCCTTCTCCTG |
| *Brs3* | F | GGCAGAAGGATGGCTGTTTGGA |
|  | R | CTCAAGTGGCTTCACGACTGCT |
| *Grin2a* | F | ACGTGACAGAACGCGAACTT |
|  | R | TCAGTGCGGTTCATCAATAACG |
| *Grin2b* | F | GCCATGAACGAGACTGACCC |
|  | R | GCTTCCTGGTCCGTGTCATC |
| *Cxcl10* | F | CCAAGTGCTGCCGTCATTTTC |
|  | R | GGCTCGCAGGGATGATTTCAA |
| *Cst7* | F | GGAGCTGTACTTGCCGAGC |
|  | R | CATGGGTGTCAGAAGTTAGGC |
| *Kcnh7* | F | CTTCGGAGAAGGCGATTGTCCT |
|  | R | CGAAGTGCTTCTTGGAACTCTGG |
| *Efna1* | F | CTTCACGCCTTTTATCTTGGGC |
|  | R | TGGGGATTATGAGTGATTTTGCC |
| *Mag* | F | CTGCCGCTGTTTTGGATAATGA |
|  | R | CATCGGGGAAGTCGAAACGG |
| *C1ql1* | F | GCACGGCCACCTATACCAC |
|  | R | CCGCATCGTAGTTGTTGCCT |
| *Gpr17* | F | CACCCTGTCAAGTCCCTCAAG |
|  | R | GTGGGCTGACTAGCAGTGG |
| *Zfp488* | F | CAGGACTCATCACCACTGTGGA |
|  | R | CCTGGAAAGCATTGCAGAGGAG |
| *Rapsn* | F | GTGGATGAAGGTGCTGGAGAAG |
|  | R | CCGAGCAGTATCAATCTGGACC |
